# Microvascular decompression for trigeminal neuralgia secondary to vertebrobasilar dolichoectasia: a systematic review and meta-analysis

**DOI:** 10.1007/s00701-026-06925-0

**Published:** 2026-05-28

**Authors:** Bardia Hajikarimloo, Ibrahim Mohammadzadeh, Salem M. Tos, Mohammad Amin Habibi, Mohammadmahdi Sabahi, Badih Adada, Hamid Borghei-Razavi

**Affiliations:** 1 Department of Neurological Surgery, University of Virginia, Charlottesville, VA USA; 2 Skull Base Research Center, Loghman-Hakim Hospital, Shahid Beheshti University of Medical Sciences, Tehran, Iran; 3 Department of Neurosurgery, Shariati Hospital, Tehran University of Medical Sciences, Tehran, Iran; 4 Department of Neurological Surgery, Pauline Braathen Neurological Center, Cleveland Clinic Florida, Weston, FL USA; 5 Cleveland Clinic Lerner College of Medicine of Case Western Reserve University, Cleveland, OH USA; 6 Neurological Surgery at CCLCM of CWRU Director of Minimally Invasive Cranial and Pituitary Surgery Program Research Director, Neuroscience Institute, Cleveland Clinic Florida Region, Cleveland, OH USA

**Keywords:** Trigeminal neuralgia, Vertebrobasilar dolichoectasia, Microvascular decompression, Systematic review, Meta-analysis

## Abstract

**Background:**

Trigeminal neuralgia (TN) caused by vertebrobasilar dolichoectasia (VBD) is a rare but particularly challenging entity. Microvascular decompression (MVD) is considered the most definitive treatment; however, outcomes in this subgroup remain incompletely characterized.

**Methods:**

We conducted a systematic review and meta-analysis following PRISMA guidelines. PubMed, Embase, Scopus, and Web of Science were searched from inception through August 22, 2025. Eligible studies reported on patients with VBD-TN undergoing MVD with extractable data on pain outcomes, recurrence, salvage interventions, or complications. Complete relief was defined as Barrow Neurological Institute (BNI)-I, while adequate relief included BNI-I to IIIb.

**Results:**

Thirteen studies involving 315 patients were analyzed. The mean age ranged from 54.0 to 67.3 years, with 57.8% (182/315) being males. The pooled initial complete pain relief rate was 95.8% (95% CI, 92.3–98.2), with sustained relief at the last follow-up in 92.6% (95% CI, 88.4–96.1). Adequate relief was nearly universal, at 99.9% (95% CI, 98.2–100%) initially and 95.9% (95% CI, 91.8–98.8%) at the last follow-up. Pain recurrence occurred in 5.5% (95% CI, 2.9–8.9%), and salvage procedures were required in 1.3% (95% CI, 0.2–3.1%). The permanent morbidity was low at 2.4% (95% CI, 0.8–4.8%). Meta-regression indicated that prior ablative procedures were associated with higher complication rates, whereas V2 involvement predicted better long-term pain control.

**Conclusion:**

MVD appears to provide effective and durable pain relief for selected patients with VBD-TN, with low permanent morbidity but a clinically meaningful overall complication burden. Given the retrospective nature of the available evidence, MVD should be considered a promising treatment option rather than a definitive standard of care.

**Supplementary Information:**

The online version contains supplementary material available at 10.1007/s00701-026-06925-0.

## Introduction

Trigeminal neuralgia (TN) is a debilitating craniofacial pain syndrome characterized by recurrent, paroxysmal, shock-like attacks along one or more branches of the trigeminal nerve [8, 17, 20]. While most cases are attributed to neurovascular compression by small arteries, most commonly the superior cerebellar artery, a minority of patients harbor compression from a dolichoectatic vertebrobasilar system [22, 31, 35]. Vertebrobasilar dolichoectasia (VBD) is an uncommon but clinically significant vasculopathy defined by elongation, tortuosity, and ectasia of the vertebral or basilar arteries [22, 31, 35]. Epidemiologic estimates suggest that VBD accounts for approximately 2–7% of TN cases, making it a rare but challenging entity for both diagnosis and surgical management [22, 31, 35].

Patients with TN secondary to VBD (VBD-TN) typically present later in life, usually in their sixth or seventh decade, and show a male predominance [7, 29, 34, 36]. The clinical course is frequently more refractory to medical therapy than idiopathic TN, and carbamazepine resistance is a common trigger for surgical referral [7, 26, 29, 34]. Surgical intervention remains the mainstay for medically refractory TN, with microvascular decompression (MVD) established as the most definitive therapy in classical cases [7, 29, 34, 36]. However, in the setting of VBD, technical difficulties arise due to the size, rigidity, and proximity of the offending artery to the brainstem [7, 29, 34, 36]. Alternative procedures, such as percutaneous balloon compression (PBC), radiofrequency thermocoagulation (RFT), or stereotactic radiosurgery (SRS), have been applied in this context; however, each carries trade-offs between durability, side effects, and invasiveness [7, 29, 34, 36].

Despite growing case series and institutional experiences, the evidence base for VBD-TN remains limited by small sample sizes, heterogeneous techniques, and variable reporting of outcomes. Most available studies are retrospective and single-center, precluding firm conclusions about long-term efficacy or safety of MVD compared with less invasive alternatives. To date, no consensus has been reached on the optimal surgical strategy for this rare but clinically significant entity. A systematic review and meta-analysis is therefore warranted to synthesize the available literature, quantify surgical outcomes, and provide evidence-based guidance for the treatment of VBD-TN.

## Materials and methods

### Objective

The objective of this systematic review and meta-analysis was to evaluate the efficacy and safety of MVD in the management of VBD-TN. Specifically, we aimed to synthesize existing evidence on surgical outcomes, including rates of pain relief, recurrence, and treatment-related complications, in order to provide an evidence-based perspective on the role of MVD in this rare but challenging clinical entity. The review was conducted in accordance with the “Preferred Reporting Items for Systematic Reviews and Meta-Analyses” (PRISMA) guidelines [21]. The study was not prospectively registered in PROSPERO or another systematic review registry.

### Search strategy

A comprehensive literature search was conducted in PubMed, Embase, Scopus, and Web of Science, spanning from database inception to August 22, 2025. The search used keywords related to “microvascular decompression”, “trigeminal neuralgia”, and “vertebrobasilar dolichoectasia/ectasia”. Detailed search strings for each database are provided in Supplementary Table S1.

### Eligibility criteria

The PICO (Population, Intervention, Comparison, Outcome) is provided in the Supplementary Table S2. The inclusion criteria were as follows. Eligible studies reported on patients with VBD-TN, confirmed by imaging and/or intraoperative findings. Patients had to be treated with MVD using any technique, including interposition or transposition methods. Studies were required to include a minimum of seven patients with VBD undergoing MVD. Eligible designs included prospective or retrospective cohort studies and case series that reported relevant outcomes. Outcomes of interest included at least one of the following: Barrow Neurological Institute (BNI) pain scores, recurrence, salvage interventions, or procedure-related complications. Only full-text articles published in English, or studies with data clearly extractable in English, were included.

The exclusion criteria were as follows. Case reports or case series with fewer than seven VBD-TN patients undergoing MVD were excluded. Reports describing vertebral artery (VA) or basilar artery (BA) compression without a confirmed diagnosis of VBD were not considered eligible. Studies with mixed cohorts in which VBD outcomes could not be separated from other etiologies were excluded. Studies were also excluded if MVD was not performed unless a distinct MVD VBD subgroup was reported. Furthermore, studies that did not provide extractable VBD-specific outcome data in event/total or convertible format were excluded. Animal studies, reviews, editorials, conference abstracts, and duplicate or overlapping cohorts were also excluded, with only the most comprehensive or latest report retained in such cases.

### Study selection process, data extraction, and risk of bias assessment

After completing the literature search, all retrieved records were uploaded into Covidence for screening and evaluation. Title and abstract screening was carried out independently by two authors, with any conflicts resolved through the assessment of a third author. Studies meeting the predefined inclusion criteria proceeded to full-text review. Two authors independently evaluated the full manuscripts, and any discrepancies were resolved by consultation with a third reviewer. Studies meeting the eligibility criteria were advanced to data extraction. This process was performed independently by two reviewers, and any conflicts were adjudicated by a third author. The list of extracted variables is provided in Supplementary Table S3. The risk of bias assessment was conducted independently by two authors using the Methodological Index for Non-Randomized Studies (MINORS) tool; any disagreements were resolved through discussion with a third reviewer [28].

Pain outcomes were classified based on the BNI pain intensity scale. Complete pain relief was defined as BNI I (pain-free without medication), whereas adequate pain relief included BNI I–IIIb (complete or functionally acceptable control, with or without medication). Pain recurrence was considered the return of trigeminal pain after an initial response, indicated by worsening to BNI ≥ IV or the need for additional treatment. Salvage intervention referred to any subsequent procedure performed after recurrence. Transient complications were events that resolved during follow-up, while permanent complications involved lasting deficits such as hearing loss, diplopia, or facial numbness (Supplementary Table S4).

### Statistical analysis

Analyses were performed in R (version 4.4.2) using the "meta" and "metafor" packages. Event-based outcomes were synthesized as proportions with 95% confidence intervals (CI). Pooled estimates were obtained using the Freeman–Tukey double-arcsine transformation and inverse-variance weighting; both fixed- and random-effects models are reported, with Hartung–Knapp adjustment applied for random-effects CIs. Heterogeneity was quantified with I2 and Cochran’s Q, and was considered noteworthy when I2 exceeded 40% or the Q test p-value was less than 0.10. When studies did not provide a specific denominator for permanent complications, the overall complication denominator was used by default.

Leave-one-out sensitivity analyses were conducted for each pooled endpoint to assess robustness. Small-study effects and publication bias were examined, when feasible (k ≥ 3), using funnel plots, Egger’s linear regression test, and the trim-and-fill method. Univariate meta-regression was performed using restricted maximum likelihood on logit-transformed proportions. Candidate moderators included mean age, sex distribution, side (right/left/bilateral), offending vessel (vertebral, basilar, or combined), prior procedures (MVD, rhizotomy, radiosurgery), trigeminal division involvement, operative approach and technique, baseline pain duration, and mean follow-up period. For each regression, coefficients, standard errors, z-statistics, p-values, τ2, and the number of studies (k) are reported, with statistical significance defined as *p* < 0.05. When continuous variables were reported as medians, they were converted to means using the method of Luo et al. to standardize parameters across studies [15].

## Results

### Study selection process

**Fig. 1:**
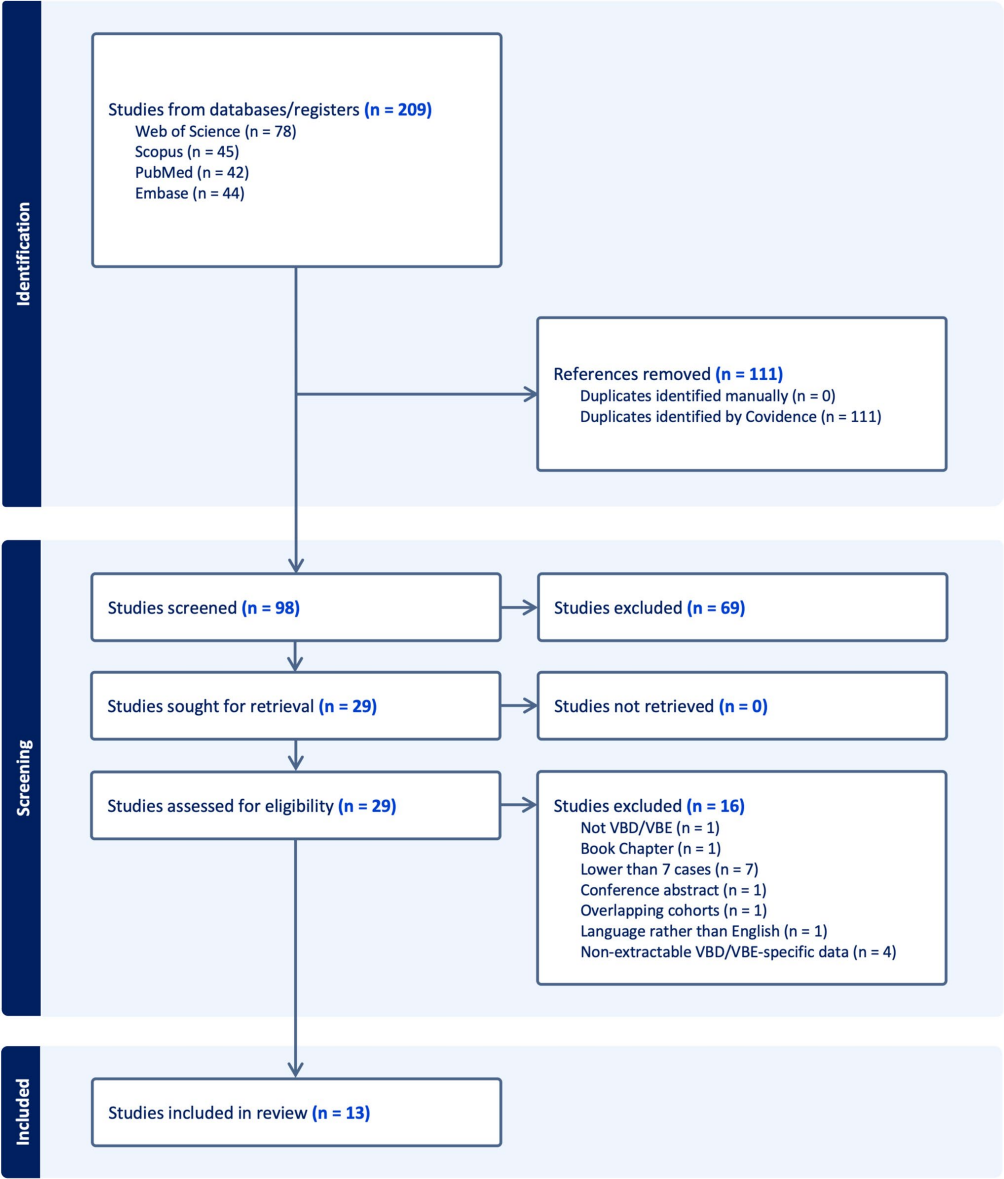
PRISMA flowchart of the current study

A total of 209 records were identified through database searches (Web of Science, *n* = 78; Scopus, *n* = 45; PubMed, *n* = 42; Embase, *n* = 44) (Fig. 1). After removing 111 duplicates using Covidence, 98 unique records remained for screening. Title and abstract screening excluded 69 studies, leaving 29 full-text articles for eligibility assessment. Of these, 16 were excluded for the following reasons: not related to vertebrobasilar dolichoectasia/elongation (*n* = 1), book chapter (*n* = 1), fewer than seven cases reported (*n* = 7), conference abstract (*n* = 1), overlapping cohorts (*n* = 1), non-English language (*n* = 1), and absence of extractable VBD-specific data (*n* = 4) [1–6, 10–14, 19, 25, 27]. Thirteen studies ultimately met the inclusion criteria and were included in the final review and quantitative synthesis [6, 7, 9, 16, 24, 26, 29, 30, 32–36].

### Risk of bias assessment

The methodological quality of the included studies was generally moderate, as assessed using the MINORS tool. None of the studies performed prospective sample size calculations, and most relied on retrospective data collection. Across domains, the aims were clearly stated, inclusion of consecutive patients was adequate, and follow-up duration and outcome assessments were generally appropriate. Total MINORS scores ranged from 10 to 13 out of 16, with one study rated as good quality, while the remainder were moderate (scores 10–12). Overall, the evidence base is limited by the non-randomized, predominantly retrospective designs; however, it demonstrates consistent reporting across key methodological domains (Supplementary Table S5).

### Baseline characteristics

Thirteen studies with 315 VBD-TN cases who had MVD were included (Table 1). The included studies were published from 2010 to 2025. The mean age ranged from 54.0 to 67.3 years, with 57.8% (182/315) males and 42.2% (133/315) females. Pain was more often left-sided in 56.5% (178/315) of cases compared to 43.5% (137/315) of right-sided cases. The vertebral artery was the most frequent offending vessel, observed in 63.8% (162/254), followed by the basilar artery in 33.9% (86/254), and combined vertebrobasilar compression in 2.4% (6/254). Secondary anterior inferior cerebellar artery involvement was frequently reported, ranging from 20 to 57% across cohorts. Mean duration of pain initiation to MVD ranged from 34.8 to 96 months. Distribution of pain branches most commonly involved V2–V3 (113/303, 37.3%), followed by isolated V2 (63/303, 20.8%) and isolated V3 (55/303, 18.2%). Less frequent patterns included isolated V1 (17/303, 5.6%), combined V1–V2 (32/303, 10.6%), and combined V1–V2–V3 (23/303, 7.6%), while V1–V3 was not reported. Among cases with detailed reporting of operative techniques (*n* = 254), interposition was the most common procedure (201/254, 79.1%), followed by transposition (53/254, 20.9%).

**Table 1:** Baseline and operative characteristics of included studies

Study	No. of Pts	Mean Age	Gender (M/F)	Side (R/L/Bi)	Offending Artery (VA/BA/VA + BA)	Prior Interventions (MVD/Rhiz/SRS)	Mean Pain Duration (m)	Trigeminal Branch							Retrosigmoid (n)	Interposition (n)	Transposition (n)
								V1	V2	V3	V1 + V2	V1 + V3	V2 + V3	V1 + V2 + V3			
Gao et al. [7]	64	64.10	37/27	30/34/0	49/15/0	0/18/3	55.2	5	11	19	6	0	19	4	64	64	0
Sun et al. [29]	26	61.00	14/12	16/10/0	16/8/2	0/1/0	38.4	1	14	1	2	0	7	1	26	26	0
Zheng et al. [36]	61	59.80	31/30	34/27/0	NA/NA/NA	0/0/0	46.8	6	11	14	6	0	21	3	61	NA	NA
Yu et al. [34]	30	63.03	21/9	8/22/0	24/6/0	0/0/0	56.13	0	6	1	5	0	15	3	30	30	0
Zhao et al. [35]	46	61.10	28/18	16/30/0	37/9/0	0/3/3	45.6	NA	NA	NA	3	0	20	7	46	22	24
Shulev et al. [26]	14	66.00	4/10	4/10/0	4/9/1	0/0/0	59.6	4	9	11	2	0	6	1	14	14	NA
Honey et al. [9]	13	67.30	10/3	5/8/0	6/7/0	0/1/1	NA	1	3	3	2	0	3	1	13	2	11
Sun et al. [30]	15	60.80	8/7	9/6/0	8/7/0	0/1/0	34.8	NA	NA	NA	NA	NA	NA	NA	15	15	0
Vanaclocha et al. [32]	8	64.88	5/3	2/6/0	0/6/2	0/2/0	NA	0	1	3	0	0	4	0	8	0	8
Ruiz-Juretschke et al. [24]	7	67.10	5/2	1/6/0	3/4/0	0/0/0	96	0	1	1	3	0	1	1	7	7	0
Ma et al. [16]	11	62.50	8/3	3/8/0	6/5/0	0/5/0	46.6	0	3	0	0	0	8	0	11	11	0
Yang et al. [33]	10	64.00	5/5	5/5/0	6/4/0	0/0/0	78.6	0	3	1	2	0	4	0	0	0	10
El-Ghandour et al. [6]	10	54.00	6/4	4/6/0	3/6/1	0/2/0	54	0	1	1	1	0	5	2	10	10	0

*NA* Not Available, *M* Male, *F* Female, *TN* Trigeminal Neuralgia, *VBD* Vertebrobasilar Dolichoectasia, *VBE* Vertebrobasilar Ectasia

### Clinical outcomes

The mean follow-up duration across studies ranged from 14.0 to 118.8 months (Table 2). Rates of initial complete pain freedom varied from 80.0% to 100%, while initial adequate pain relief was consistently high at 92.3% to 100%. At last follow-up, complete pain-free rates ranged from 78.3% to 100%, and adequate pain relief ranged from 87.0% to 100%. Pain recurrence was reported in 0% to 14.3% of patients, and salvage interventions in 0% to 14.3%. Reported overall complication rates ranged widely from 0% to 53.1%, with transient complications occurring in 0% to 50% and permanent complications in 0% to 23.1%.

**Table 2:** Outcome characteristics (Percentages) — MVD for trigeminal neuralgia due to VBD/VBE

Study	Mean FU (months)	Initial complete pain-free (%)	Initial adequate pain relief (%)	Last FU complete pain-free (%)	Last FU adequate pain relief (%)	Pain Recurrence (%)	Salvage Intervention (%)	Overall Complication (%)	Transient Complication (%)	Permanent Complication (%)
Gao et al. [7]	46.80	95.3%	100%	80.6%	89.1%	12.5%	NA	53.1%	43.8%	3.1%
Sun et al. [29]	22.00	100%	100%	100%	100%	0%	0%	3.8%	0%	3.8%
Zheng et al. [36]	24.50	83.6%	93.4%	NA	NA	9.8%	NA	27.9%	NA	NA
Yu et al. [34]	76.67	100%	100%	90%	90%	10%	10%	0%	0%	0%
Zhao et al. [35]	61.10	93.5%	100%	78.3%	87%	13%	NA	30.4%	17.4%	6.5%
Shulev et al. [26]	14.00	100%	100%	100%	100%	0%	0%	50%	50%	0%
Honey et al. [9]	118.80	92.3%	92.3%	88.9%	88.9%	8.3%	7.7%	38.5%	23.1%	23.1%
Sun et al. [30]	29.80	100%	100%	100%	100%	0%	0%	13.3%	0%	13.3%
Vanaclocha et al. [32]	56.50	100%	100%	100%	100%	0%	0%	25%	12.5%	12.5%
Ruiz-Juretschke et al. [24]	51.00	100%	100%	85.7%	100%	14.3%	14.3%	14.3%	14.3%	0%
Ma et al. [16]	22.00	81.8%	100%	NA	100%	0%	0%	45.5%	45.5%	0%
Yang et al. [33]	NA	80%	100%	NA	100%	0%	0%	10%	10%	0%
El-Ghandour et al. [6]	93.60	80%	100%	80%	100%	0%	0%	40%	40%	0%

*BNI* Barrow Neurological Institute pain scale; Adequate pain relief = BNI I–IIIb, *FU* Follow-Up, *NA* Not Available

### Meta-analysis of outcomes

**Fig. 2:**
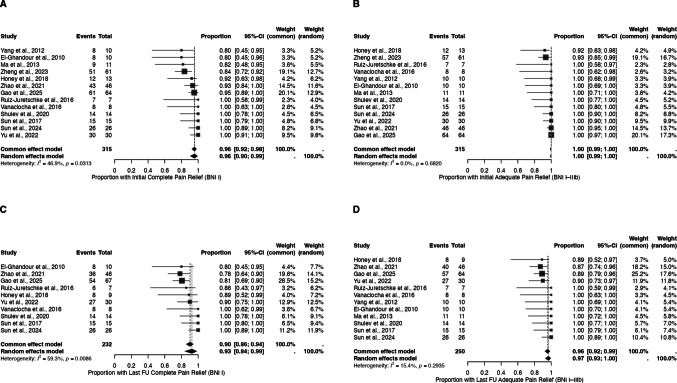
Pain outcomes following MVD for VBD-TN: **A** initial complete pain relief, **B** initial adequate pain relief, **C** last follow-up complete pain relief, **D** last follow-up adequate pain relief

Across 13 studies comprising 315 patients, the pooled rate of initial complete pain relief after MVD was 95.8% (95% CI, 90.5–99.3%; I2 = 0.5, *p* = 0.0313) (Fig. 2A). The rate of initial adequate pain relief was 99.9% (95% CI, 98.7–100.0%; I2 = 0.0%, *p* = 0.682) (Fig. 2B). At last follow-up, which included 232 patients from 10 studies, complete pain relief persisted in 92.6% (95% CI, 83.9–98.5%; I2 = 0.6, *p* = 0.009) (Fig. 2C). Adequate pain relief was maintained at the last follow-up in 95.9% (95% CI, 92.4–98.5%) of 250 patients across 12 studies (I2 = 0.2, *p* = 0.294) (Fig. 2D).

**Fig. 3:**
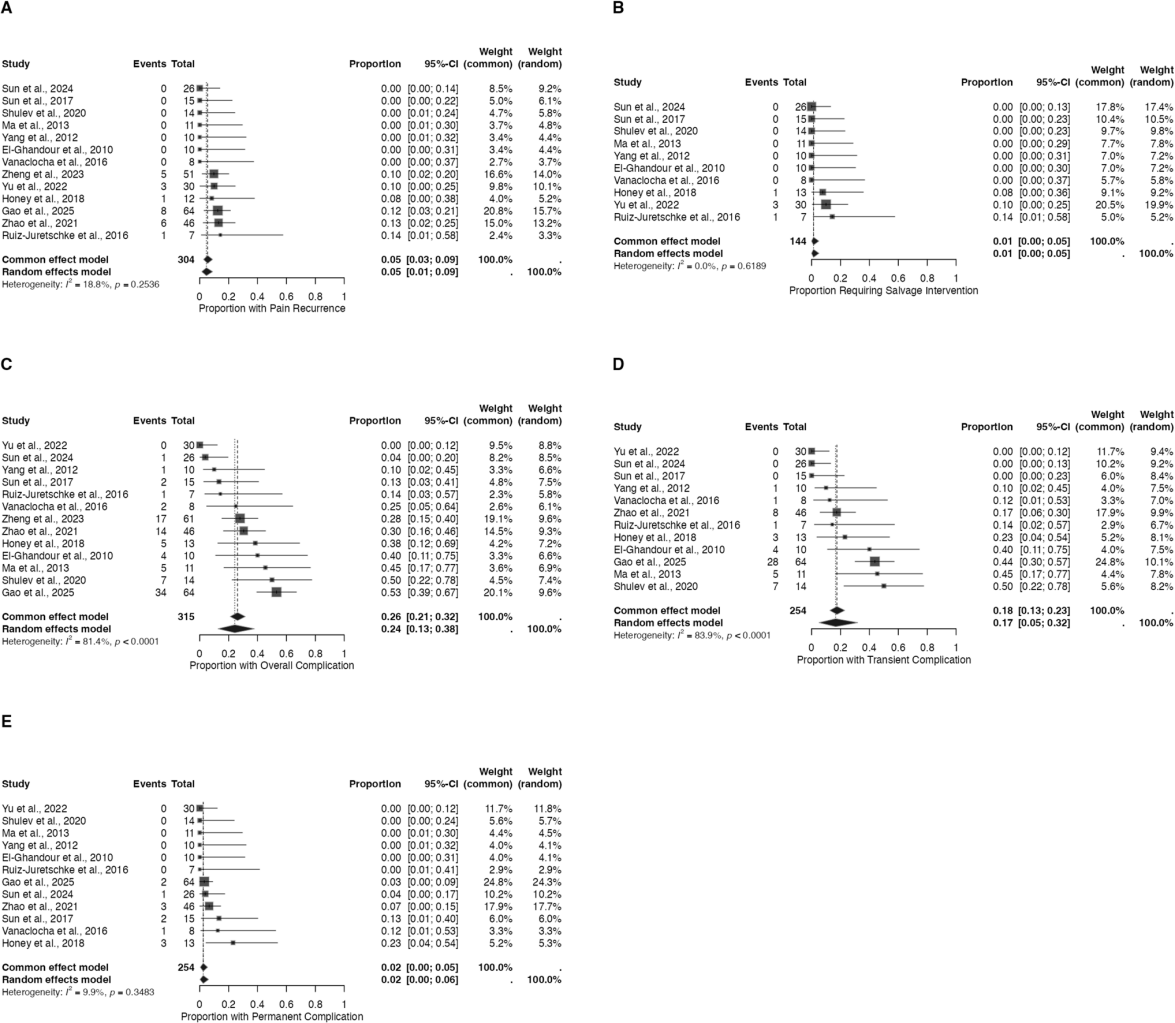
Secondary outcomes following MVD for VBD-TN: **A** pain recurrence, **B** salvage interventions, **C** overall complications, **D** transient complications, **E** permanent complications

The pooled pain recurrence rate was 5.5% (95% CI, 2.7–8.9%; I2 = 0.2, *p* = 0.254) (Fig. 3A). Salvage interventions were rarely required, reported in only 1.3% (95% CI, 0.0–5.0%) of 144 patients across 10 studies (I2 = 0.0, *p* = ^0.619)^ (Fig. 3B). Regarding safety, the pooled incidence of overall complications was 24.3% (95% CI, 12.7–37.8%; I2 = 0.8, *p* < 0.001) (Fig. 3C). Transient complications were reported in 16.7% (95% CI, 5.1–32.0%; I2 = 0.8%, *p* < 0.001) (Fig. 3D), while permanent complications were uncommon, occurring in 2.4% (95% CI, 0.4–5.4%; I2 = 0.1, *p* = 0.348) (Fig. 3E).

Exploratory meta-regression analyses suggested that prior SRS exposure may be associated with reduced likelihood of last follow-up complete pain relief (estimate = –0.1430, *p* = 0.0550, borderline significance), while a higher proportion of V2 involvement predicted greater rates of last follow-up complete pain relief (estimate = 0.0464, *p* = 0.0488). Additionally, studies with a higher proportion of patients who had undergone prior rhizotomy showed significantly higher rates of overall complications (estimate = 0.0311, *p* = 0.0417) (Supplementary Table S6).

### Sensitivity analysis

Leave-one-out sensitivity analyses showed that the findings for initial complete pain relief (96%; 95% CI, 92–98%) and initial adequate pain relief (100%; 95% CI, 99–100%) were robust and remained consistent (Supplementary Fig. 1A–B). Results for the last follow-up, complete pain relief (90%; 95% CI, 86–94%), were not entirely consistent, as excluding certain studies slightly changed the pooled estimate, while last follow-up adequate pain relief (96%; 95% CI, 92–99%) remained consistent (Supplementary Fig. 1C–D).

The pooled estimates for pain recurrence (5%; 95% CI, 3–9%) and salvage interventions (1%; 95% CI, 0–5%) remained consistent (Supplementary Fig.  2 A and B). For complications, the pooled overall complication rate varied more widely (20.0–30.9%), suggesting some influence by individual studies, though the direction of effect remained consistent (Supplementary Fig.  3 C). Transient complications showed similar variability (10.5–21.8%), reflecting moderate sensitivity (Supplementary Fig. 3D). By contrast, permanent complications remained very low and stable (1.8–3.1%), supporting the robustness of this outcome (Supplementary Fig. 3E).

### Publication bias

Egger’s test did not indicate significant publication bias for initial complete pain relief (*p* = 0.93), initial adequate pain relief (*p* = 0.64), last follow-up complete pain relief (*p* = 0.21), salvage interventions (*p* = 0.94), overall complications (*p* = 0.60), transient complications (*p* = 0.76), or permanent complications (*p* = 0.58). In contrast, significant small-study effects were observed for adequate pain relief at the last follow-up (*p* = 0.027) and pain recurrence (*p* = 0.039).

Trim-and-fill analysis imputed no missing studies for most outcomes but adjusted the pooled estimate for last follow-up adequate pain relief after imputing five studies (random-effects pooled rate 92.8%, 95% CI 86.7–97.5%) and for pain recurrence after imputing four studies (random-effects pooled rate 7.7%, 95% CI 3.1–13.6%). These adjustments did not materially alter the interpretation of the main findings.

## Discussion

In this meta-analysis of 13 studies involving 315 patients with VBD-TN, MVD achieved initial complete pain relief in 95.8% of cases and adequate relief in 99.9% of cases. Importantly, complete pain relief reflected BNI I only, whereas adequate pain relief included BNI I–IIIb and therefore represented a broader endpoint that could include patients with residual pain or ongoing medication use. At the final follow-up (14–119 months), complete pain freedom persisted in 92.6% of patients and adequate relief in 95.9%, suggesting favorable durability in the available literature. These pain relief rates appear favorable in the context of MVD outcomes reported for TN more broadly; however, VBD-TN is technically more challenging because dolichoectatic vertebrobasilar arteries are larger, stiffer, and more difficult to mobilize, potentially increasing perioperative risk [7, 30, 34]. Pain recurrence was relatively rare (5.5%), and the need for salvage procedures was low (1.3%). Complications occurred in 24.3% of patients, which represents a clinically meaningful morbidity burden, although most events were transient (16.7%) and permanent morbidity remained low (2.4%). Therefore, the durable pain relief observed after MVD should be balanced against its perioperative risks, particularly when counseling older patients, patients with comorbidities, or those with complex vertebrobasilar anatomy.

Notably, meta-regression identified several factors influencing outcomes: prior SRS was associated with lower rates of long-term complete relief (estimate = –0.143, *p* = 0.055, borderline significance), while a higher proportion of V2 division involvement predicted better long-term pain relief (estimate = 0.046, *p* = 0.0488). Conversely, patients with a more extensive history of prior rhizotomy showed higher overall complication rates (estimate = 0.031, *p* = 0.0417). Given the limited number of studies, these meta-regression findings should be interpreted cautiously as exploratory signals rather than definitive evidence of predictors of outcome (Table 3).

**Table 3:** Summary of pooled outcomes

Endpoint	k	N	Fixed	I^2^	p-value
Initial complete pain relief	13	315	95.8% (90.5–99.3%)	0.5%	0.0313
Initial adequate pain relief	13	315	99.9% (98.7–100.0%)	0.0%	0.682
Last FU complete pain relief	10	232	92.6% (83.9–98.5%)	0.6%	0.009
Last FU adequate pain relief	12	250	95.9% (92.4–98.5%)	0.2%	0.294
Pain recurrence	13	304	5.5% (2.7–8.9%)	0.2%	0.254
Salvage intervention	10	144	1.3% (0.0–5.0%)	0.0%	0.619
Overall complication	13	315	24.3% (12.7–37.8%)	0.8%	< 0.001
Transient complication	12	254	16.7% (5.1–32.0%)	0.8%	< 0.001
Permanent complication	12	254	2.4% (0.4–5.4%)	0.1%	0.348

k = number of studies; N = total patients; FU = follow-up; I^2^ = heterogeneity index; p-value = significance level; % () = pooled rate with 95% confidence interval

These findings align with series reporting excellent outcomes of MVD in carefully selected VBD-TN cases, but they also underscore the heterogeneity in complication profiles [6, 7, 9, 16, 24, 26, 29, 30, 32–36]. In contrast to classical TN, where superior cerebellar artery decompression typically carries lower morbidity, the presence of ectatic vertebrobasilar arteries introduces unique technical challenges [9]. Compared with less invasive options such as SRS, percutaneous balloon compression (PBC), or radiofrequency thermocoagulation (RFT), our pooled data suggest that MVD achieves superior and more durable pain control, though at the cost of higher perioperative risk.

Zhao et al. compared outcomes of MVD and two-isocenter Gamma Knife radiosurgery (GKRS) in 80 patients with VBD-TN [35]. They reported significantly higher initial favorable outcomes with MVD (97.8% vs. 78.9%) and better long-term durability, with 82.6% of MVD patients versus 58.8% of GKRS patients maintaining pain control at last follow-up (*p* = 0.003) [35]. Kaplan–Meier analysis similarly demonstrated superior 7-year pain-free rates after MVD (74.2% vs. 47.2%) [35]. Gao et al. conducted a retrospective cohort study of 107 patients with VBD-TN, comparing 64 who underwent MVD and 43 who underwent PBC [7]. Both groups achieved excellent initial pain relief; however, recurrence at final follow-up was lower after MVD (12.5%) than after PBC (20.9%), although the difference was not statistically significant (*p* = 0.242) [7]. PBC was associated with significantly higher rates of facial numbness, dry eye, and masticatory weakness, whereas MVD carried risks of craniotomy-related complications, including infection (4.7%), CSF leakage (3.1%), and hearing loss (7.8%) [7].

Several studies have evaluated the role of SRS for patients with VBD-TN who are poor candidates for MVD or in whom vascular anatomy renders decompression particularly high-risk. Park et al. treated 20 patients with GKRS and reported adequate initial pain relief in 75%. However, durability was limited, with pain-free rates of 53% at 1 year, 38% at 2 years, and 10% at 5 years. These outcomes were significantly inferior to those of non-VBD TN controls [22]. In contrast, Tuleasca et al. prospectively followed 29 patients with megadolichobasilar compression. They found initial complete pain relief in 100% of cases, with actuarial maintenance of pain freedom at 79% and 76% at 1 and 2 years, respectively [31]. However, 24% of patients recurred, and 13% developed mild facial numbness [31]. More recently, Mori et al. reported four contemporary cases using 80 Gy to the retrogasserian portion, all of which achieved remission within 10 months [18]. One case recurred at 41 months but was successfully retreated, and notably, no adverse effects were observed [18]. Collectively, these data suggest that SRS can achieve meaningful pain relief in VBD-TN, with low morbidity; however, recurrence rates remain higher, and long-term durability is lower compared to MVD.

Several studies have investigated clinical or anatomical predictors of outcomes following MVD in VBD-TN. Honey et al. emphasized that mobilization of atherosclerotic and stiff dolichoectatic arteries remains technically challenging, with outcomes depending heavily on successful vessel transposition [9]. Vanaclocha et al. highlighted that all their patients had involvement of the V2 and/or V3 divisions, suggesting that trigeminal distribution may influence surgical candidacy, though all achieved pain freedom with basilar artery repositioning and dural scarring techniques [32]. Sun et al. found that patients were typically older males with hypertension and stroke history. These factors characterized the VBD subgroup but did not preclude excellent results, as all 15 patients remained pain-free at a mean of 29.8 months [29]. More recently, Yu et al. demonstrated that VBD-TN patients were more likely to be male, hypertensive, and left-sided, and that outcomes after interposition MVD were comparable to non-VBD TN, with recurrences in only 10% over 6 years [34]. Similarly, Zheng et al. observed that direct versus indirect compression patterns did not significantly alter outcomes when a stepwise decompression strategy was used [36]. Collectively, these data suggest that male sex, hypertension, and V2/V3 involvement are common clinical features in VBD-TN. At the same time, surgical technique (interposition vs. transposition vs. stepwise decompression) and prior interventions appear to be more relevant in predicting long-term success.

Transposition was not a uniform operation across the included studies, encompassing sling-to-dura mobilization, caudal withdrawal of the proximal vertebral artery, ventrocranial transposition with petrous-bone anchoring, and basilar artery repositioning with clival dural-scar fixation [9, 32, 33, 35]. Because each sub-technique is reported by only a single small cohort, sub-technique comparison is not feasible at the study level. Moreover, transposition is often anatomically infeasible when the dolichoectatic basilar artery is firmly embedded against the pons with short brainstem perforators, in which case interposition, sometimes with Teflon placed both anterior and posterior to the trigeminal nerve, is the only safe option. Interposition and transposition are therefore better viewed as complementary, anatomy-driven strategies than as competing alternatives, and any apparent advantage of transposition likely reflects selection of more favorable anatomy.

The slight variability observed in the sensitivity analysis for last-follow-up complete pain relief probably results from differences among excluded studies in several key aspects. Studies with smaller sample sizes or primarily involving vertebral-artery compression tend to show lower long-term success, likely due to more complex anatomy and limited decompression space. Moreover, shorter follow-up periods and inconsistent definitions of “complete pain-free” status may have also caused fluctuations in the pooled estimates. Overall, these factors indicate that anatomical complexity, surgical approach, and follow-up duration significantly impact the consistency of long-term pain relief results.

A recent publication by Pour-Rashidi et al. provided a significant overview of VBD–TN and hemifacial spasm, combining institutional experience with a comprehensive review of the literature [23]. Our study builds upon and enhances that valuable work by focusing specifically on MVD for VBD-TN, employing a quantitative meta-analytic approach. By synthesizing data from eligible cohorts with measurable outcomes and applying rigorous statistical methods, including pooled estimates, sensitivity analyses, and meta-regression, we aimed to offer a data-driven perspective on the durability of pain relief, recurrence, and complications. While the prior study provides a broad clinical and qualitative foundation, our analysis expands these insights through quantitative synthesis to support evidence-based decision-making in this rare and challenging condition.

Our findings support MVD as the most effective treatment for VBD-TN, with pooled rates of initial pain relief over 95% and long-term durability above 90%, despite the technical challenges posed by dolichoectatic vessels. For clinical practice, this indicates that carefully selected patients, typically those who are medically refractory and fit for surgery, should be offered MVD as the first-line treatment when possible, provided the procedure is performed at experienced centers with expertise in skull base vascular mobilization. Alternative options such as SRS or PBC may be reserved for patients with prohibitive surgical risk or unfavorable anatomy, as these have lower durability and higher recurrence rates. Importantly, surgical planning should incorporate predictors identified in our meta-regression, including prior destructive procedures, trigeminal division involvement, and the choice between interposition and transposition techniques, to maximize safety and effectiveness.

This study has several limitations. The evidence base remains limited by the small number of included studies (k = 13), all of which were non-randomized and predominantly retrospective single-center series. This design increases the risk of selection bias, as patients selected for MVD were likely surgically fit and treated at specialized centers with experience in complex neurovascular decompression. Therefore, the favorable pooled outcomes may not be fully generalizable to all patients with VBD-TN, particularly those with higher operative risk or unfavorable vascular anatomy. Outcome definitions varied across studies, especially regarding “adequate relief” and complication reporting, which may have introduced misclassification bias. Follow-up duration varied widely, ranging from 14 to 119 months, potentially leading to underestimation of late recurrences. Important clinical and anatomical variables, including the severity of arterial ectasia, surgical experience, imaging protocols, and perioperative management, were inconsistently reported, limiting more detailed subgroup analyses. Although meta-regression provided exploratory insights, it was constrained by the small number of studies for each covariate and should be interpreted cautiously. The pooled "transposition" category subsumes four conceptually distinct sub-techniques each reported by a single cohort, and the choice between interposition and transposition was anatomy-driven rather than randomly assigned, both of which limit causal interpretation of technique-related associations.

Publication bias cannot be fully excluded, and some studies relied on intraoperative assessment rather than standardized high-resolution MRI to confirm neurovascular conflict. Although I2 values were low across most endpoints, this should not be interpreted as absence of clinical heterogeneity, as the small number of studies, sparse event counts, variable follow-up, and proportion-based data structure may have limited the ability to detect true between-study variability. Although meta-regression provided exploratory insights, these analyses were likely underpowered because of the limited number of included studies and the small number of studies available for each covariate. Therefore, these associations should be interpreted cautiously and should not be considered definitive predictors of outcome. In addition, this review was not prospectively registered in PROSPERO or another systematic review registry, which may limit protocol transparency.

Future research should focus on prospective, multicenter registries with standardized outcome reporting to address the limitations of small, heterogeneous single-center series. Comparative effectiveness studies between MVD and alternative modalities, such as SRS or percutaneous procedures, are necessary, ideally stratified by vascular anatomy and surgical technique. Advanced imaging that better characterizes neurovascular conflict and predicts surgical difficulty may assist in preoperative planning and patient selection. Additionally, innovations in vascular mobilization, endoscopic visualization, and intraoperative neuromonitoring should be systematically evaluated to enhance safety in this technically demanding population. Given the rarity of VBD-TN, international collaboration is crucial for developing adequately powered datasets that can inform consensus guidelines and enhance evidence-based treatment algorithms.

## Conclusion

This systematic review and meta-analysis suggests that MVD may provide effective and durable pain relief for selected patients with VBD-TN, with low permanent morbidity in the available literature. However, the evidence is limited by small, retrospective, mostly single-center studies without randomized or high-quality comparative data. Therefore, MVD should be considered a promising treatment option for surgically fit patients, while SRS or percutaneous procedures may remain appropriate for selected high-risk cases. Prospective comparative studies are needed to better define its role.

## Supplementary Information

Below is the link to the electronic supplementary material.

ESM 1 Supplementary Material 1 (PDF 104 KB)

ESM 2 Supplementary Material 2 (PDF 1.32 MB)

## Data Availability

“The data supporting this study's findings are available from the corresponding author upon reasonable request.”

## Declarations

### AI usage

During manuscript preparation, the authors used Anthropic's Claude and OpenAI's ChatGPT for language refinement. These tools were not used to design the study, conduct or interpret analyses, generate data, or produce citations. All analyses were performed by the authors in R (v4.4.2). All references were independently verified by the authors against primary sources. All AI-assisted text was reviewed and verified by the authors, who take full responsibility for the manuscript's content. No confidential patient information was shared.

“This manuscript complies with all instructions to authors. All authors meet authorship criteria and have approved the final manuscript. This manuscript has not been published and is not under consideration elsewhere. A PRISMA checklist was used and is included with the submission.”
