# Supplementary material for: Microvascular decompression for trigeminal neuralgia secondary to vertebrobasilar dolichoectasia: a systematic review and meta-analysis
Source: Acta Neurochir (Wien). 2026 May 28;168(1):168. doi: 10.1007/s00701-026-06925-0 (PMC13415661; doi:10.1007/s00701-026-06925-0)
Supplement: Supplementary file 2 — Supplementary Material 2 (PDF 1.32 MB) [file 701_2026_6925_MOESM2_ESM.pdf]

Supplementary File

Microvascular Decompression for Trigeminal Neuralgia Secondary to Vertebrobasilar Dolichoectasia: A Systematic Review and Meta-Analysis

Supplementary Table S1: Search strategies for various databases.	
Database	Search strategy
PubMed	("Decompression, Surgical"[Mesh] OR "microvascular decompression"[tiab] OR (microvascular[tiab] AND decompression[tiab]) OR MVD[tiab])<br>AND ("Trigeminal Neuralgia"[Mesh] OR "trigeminal neuralgia"[tiab] OR "tic douloureux"[tiab])<br>AND ("vertebrobasilar ectasia"[tiab] OR "vertebrobasilar dolichoectasia"[tiab] OR "vertebrobasilar dolicho-ectasia"[tiab]<br>OR "basilar artery dolichoectasia"[tiab] OR "basilar dolichoectasia"[tiab] OR "basilar artery ectasia"[tiab]<br>OR "vertebral artery dolichoectasia"[tiab] OR "vertebral dolichoectasia"[tiab] OR "vertebral artery ectasia"[tiab]<br>OR "megadolichobasilar"[tiab]<br>OR ((vertebral[tiab] OR basilar[tiab] OR vertebrobasilar[tiab]) AND (ectasia[tiab] OR dolichoectasia[tiab])))
Embase	('microvascular decompression'/exp OR 'microvascular decompression':ti,ab OR mvd:ti,ab OR 'surgical decompression':ti,ab OR 'neurovascular decompression':ti,ab OR 'microsurgical decompression':ti,ab)<br>AND ('trigeminal neuralgia'/exp OR 'trigeminal neuralgia':ti,ab OR 'tic douloureux':ti,ab)<br>AND ('vertebrobasilar ectasia':ti,ab OR 'vertebrobasilar dolichoectasia':ti,ab OR 'vertebrobasilar dolicho-ectasia':ti,ab<br>OR 'basilar artery dolichoectasia':ti,ab OR 'basilar dolichoectasia':ti,ab OR 'basilar artery ectasia':ti,ab<br>OR 'vertebral artery dolichoectasia':ti,ab OR 'vertebral dolichoectasia':ti,ab OR 'vertebral artery ectasia':ti,ab<br>OR megadolichobasilar:ti,ab<br>OR ((vertebral:ti,ab OR basilar:ti,ab OR vertebrobasilar:ti,ab) AND (ectasia:ti,ab OR dolichoectasia:ti,ab)))
Scopus	(TITLE-ABS-KEY("microvascular decompression" OR MVD OR "surgical decompression" OR "neurovascular decompression" OR "microsurgical decompression"))<br>AND (TITLE-ABS-KEY("trigeminal neuralgia" OR "tic douloureux"))<br>AND (TITLE-ABS-KEY("vertebrobasilar ectasia" OR "vertebrobasilar dolichoectasia" OR "vertebrobasilar dolicho-ectasia"<br>OR "basilar artery dolichoectasia" OR "basilar dolichoectasia" OR "basilar artery ectasia"<br>OR "vertebral artery dolichoectasia" OR "vertebral dolichoectasia" OR "vertebral artery ectasia"<br>OR megadolichobasilar<br>OR ((vertebral OR basilar OR vertebrobasilar) AND (ectasia OR dolichoectasia))))
WOS	TS=("microvascular decompression" OR MVD OR "surgical decompression" OR "neurovascular decompression" OR "microsurgical decompression")<br>AND TS=("trigeminal neuralgia" OR "tic douloureux")<br>AND TS=("vertebrobasilar ectasia" OR "vertebrobasilar dolichoectasia" OR "vertebrobasilar dolicho-ectasia"<br>OR "basilar artery dolichoectasia" OR "basilar dolichoectasia" OR "basilar artery ectasia"<br>OR "vertebral artery dolichoectasia" OR "vertebral dolichoectasia" OR "vertebral artery ectasia"<br>OR megadolichobasilar<br>OR ((vertebral OR basilar OR vertebrobasilar) AND (ectasia OR dolichoectasia)))
August 22, 2025	

Supplementary Table S2. PICO framework	
Population (P)	Patients with trigeminal neuralgia specifically caused by vertebrobasilar dolichoectasia/ectasia (VBD/VBE) confirmed by neuroimaging and/or intraoperative findings.
Intervention (I)	Microvascular decompression (MVD) (including interposition, sling, or transposition techniques) performed for TN secondary to VBD/VBE.
Comparison (C)	No comparator required. If available, comparisons may include alternative treatments (radiosurgery, percutaneous procedures, medical therapy).
Outcome (O)	Primary: Pain relief outcomes (BNI I, BNI I–IIIb, initial response, long-term pain-free rates, recurrence).<br>Secondary: Complications (cranial nerve deficits, vascular injury, adverse events), durability of relief, need for salvage interventions.

Supplementary Table S3. Extracted variables	
Baseline	Outcome
DOI<br>Study<br>Year<br>Country<br>Design<br>Full List Of Participant Hospitals<br>No Pts<br>Mean Age<br>No Male<br>No Female<br>No Right Sided<br>No Left Sided<br>No Bilateral Sided<br>No Offending Artery Vertebral Artery<br>No Offending Artery Basilar Artery<br>No Offending Artery Vertebral And Basilar Artery<br>No Prior MVD<br>No Prior Rhizotomy<br>No Prior SRS<br>Mean Duration Pain<br>No V1 Trigeminal<br>No V2 Trigeminal<br>No V3 Trigeminal<br>No V1 and V2 Trigeminal<br>No V1 and V3 Trigeminal<br>No V2 and V3 Trigeminal<br>No V1 and V2 and V3 Trigeminal<br>No Retrosigmoid Approach<br>No Subtemporal Approach<br>No Transpetrosal Approach<br>No Other Approach<br>No Technique Interposition<br>No Technique Transposition<br>No Technique Sling<br>No Technique Teflon Padding<br>Mean Operative Time<br>No Intraoperative Monitoring	Mean Follow Up<br>Event Initial Complete Pain Relief BNI I<br>Total Initial Complete Pain Relief BNI I<br>Event Initial Adequate Pain Relief BNI I to IIIb<br>Total Initial Adequate Pain Relief BNI I to IIIb<br>Event Last FU Complete Pain Relief BNI I<br>Total Last FU Complete Pain Relief BNI I<br>Event Last FU Adequate Pain Relief BNI I to IIIb<br>Total Last FU Adequate Pain Relief BNI I to IIIb<br>Mean Time To Pain Relief BNI I IIIb<br>Event Pain Recurrence<br>Total Pain Recurrence<br>Mean Time To Pain Recurrence<br>Event Salvage Intervention<br>Total Salvage Intervention<br>Event Overall Complication<br>Total Overall Complication<br>Event Transient Complication<br>Total Transient Complication<br>Event Permanent Complication<br>Total Permanent Complication<br>Specify Complication

Supplementary Table S4. Definition of outcomes	
Initial complete pain relief (BNI I)	Complete resolution of trigeminal neuralgia pain without the need for medication, measured at the first follow-up after microvascular decompression.
Initial adequate pain relief (BNI I–IIIb)	Achievement of complete or partial pain relief allowing acceptable daily function, with or without medication use, corresponding to BNI pain intensity scores I to IIIb at the initial post-treatment follow-up.
Last follow-up complete pain relief (BNI I)	Proportion of patients maintaining BNI I (pain-free without medication) at the last available follow-up.
Last follow-up adequate pain relief (BNI I–IIIb)	Proportion of patients maintaining BNI I–IIIb (complete or acceptable pain control) at the last available follow-up.
Pain recurrence	Return of trigeminal neuralgia pain following an initial period of adequate pain relief, as defined by an increase in BNI pain intensity score (≥IV) or the need for additional medication or surgical intervention.
Salvage intervention	Requirement for additional procedures (e.g., repeat SRS, microvascular decompression, percutaneous rhizotomy, or other surgical intervention) after initial SRS failure or pain recurrence.
Overall complication	Total number of patients experiencing at least one perioperative or postoperative complication.
Transient complication	Complications that resolved within the follow-up period (e.g., transient cranial nerve deficits, temporary hearing loss, transient CSF leak).
Permanent complication	Detailed description of the type of complication reported (e.g., hearing loss, diplopia, facial numbness, CSF leak, infection, vascular injury, cerebellar infarct).

Supplementary Table S5. Risk of bias assessment of the included studies										
Study	Clearly Stated Aim	Consecutive Patient Inclusion	Prospective Data Collection	Appropriateness of Endpoints	Unbiased Outcome Assessment	Adequate Follow-Up Duration	Loss to Follow-Up <5%	Prospective Sample Size Calculation	Total Score (/16)	Quality Rating
Gao 2025	2	2	0	2	2	2	2	0	12	Moderate
Sun 2024	2	2	0	2	2	2	2	0	12	Moderate
Zheng 2023	2	2	0	2	2	2	2	0	12	Moderate
Yu 2022	2	2	0	2	2	2	2	1	13	Good
Zhao 2021	2	2	0	2	2	2	2	0	12	Moderate
Shulev 2020	2	2	0	2	2	2	1	0	11	Moderate
Honey 2018	2	2	0	2	2	2	2	0	12	Moderate
Sun 2017	2	2	0	2	2	2	1	0	11	Moderate
Vanaclocha 2016	2	1	0	2	2	2	1	0	10	Moderate
Ma 2013	2	2	0	2	2	2	1	0	11	Moderate
El-Ghandour 2010	2	2	0	2	2	2	2	0	12	Moderate
Yang 2012	2	2	0	2	2	2	2	0	12	Moderate
Ruiz-Juretschke 2016	2	2	0	2	2	2	2	0	12	Moderate

Supplementary Table S6. Meta-regression results				
Outcome	Modifier	Estimate	P_value	k
BNI Initial Complete	Mean.Age	0.1189	0.1000	13
BNI Initial Complete	Mean.Duration.Pain	-0.0095	0.6710	11
BNI Initial Complete	Mean.Follow.Up	0.0016	0.8770	12
BNI Initial Complete	male_percentage	0.0040	0.8840	13
BNI Initial Complete	female_percentage	-0.0040	0.8840	13
BNI Initial Complete	right_percentage	-0.0124	0.5660	13
BNI Initial Complete	left_percentage	0.0124	0.5660	13
BNI Initial Complete	va_percentage	0.0139	0.2760	12
BNI Initial Complete	ba_percentage	-0.0202	0.1990	12
BNI Initial Complete	vaba_percentage	-0.0071	0.8870	12
BNI Initial Complete	prior_rhizotomy_percentage	-0.0050	0.7980	13
BNI Initial Complete	prior_srs_percentage	0.1367	0.0743	13
BNI Initial Complete	v1_percentage	0.0335	0.4830	11
BNI Initial Complete	v2_percentage	0.0242	0.3180	11
BNI Initial Complete	v3_percentage	0.0190	0.3020	11
BNI Initial Complete	v1v2_percentage	0.0079	0.8210	12
BNI Initial Complete	v2v3_percentage	-0.0195	0.3360	12
BNI Initial Complete	v1v2v3_percentage	0.0183	0.6780	12
BNI Initial Complete	retrosigmoid_percentage	0.0098	0.3030	13
BNI Initial Complete	otherapproach_percentage	-0.0098	0.3030	13
BNI Initial Complete	interposition_percentage	0.0055	0.4310	12
BNI Initial Complete	transposition_percentage	-0.0050	0.4890	11
BNI Initial Adequate	Mean.Age	0.0106	0.9130	13
BNI Initial Adequate	Mean.Follow.Up	0.0000	0.9980	12
BNI Initial Adequate	male_percentage	0.0002	0.9950	13
BNI Initial Adequate	female_percentage	-0.0002	0.9950	13
BNI Initial Adequate	right_percentage	-0.0072	0.7550	13

BNI Initial Adequate	left_percentage	0.0072	0.7550	13
BNI Initial Adequate	va_percentage	0.0235	0.1990	12
BNI Initial Adequate	ba_percentage	-0.0351	0.1340	12
BNI Initial Adequate	vaba_percentage	-0.0209	0.7150	12
BNI Initial Adequate	prior_rhizotomy_percentage	0.0159	0.5460	13
BNI Initial Adequate	prior_srs_percentage	0.0576	0.6290	13
BNI Initial Adequate	v1_percentage	-0.0099	0.8430	11
BNI Initial Adequate	v2_percentage	0.0146	0.5580	11
BNI Initial Adequate	v3_percentage	-0.0023	0.9110	11
BNI Initial Adequate	v1v2_percentage	-0.0120	0.7530	12
BNI Initial Adequate	v2v3_percentage	0.0113	0.6710	12
BNI Initial Adequate	v1v2v3_percentage	0.0329	0.6150	12
BNI Initial Adequate	retrosigmoid_percentage	0.0008	0.9560	13
BNI Initial Adequate	otherapproach_percentage	-0.0008	0.9560	13
BNI Initial Adequate	interposition_percentage	0.0078	0.4130	12
BNI Initial Adequate	transposition_percentage	-0.0082	0.3980	11
BNI LastFU Complete	Mean.Age	0.0424	0.5700	10
BNI LastFU Complete	Mean.Duration.Pain	-0.0009	0.9630	8
BNI LastFU Complete	Mean.Follow.Up	-0.0092	0.4260	10
BNI LastFU Complete	male_percentage	-0.0172	0.5780	10
BNI LastFU Complete	female_percentage	0.0172	0.5780	10
BNI LastFU Complete	right_percentage	0.0046	0.8460	10
BNI LastFU Complete	left_percentage	-0.0046	0.8460	10
BNI LastFU Complete	va_percentage	-0.0144	0.1810	10
BNI LastFU Complete	ba_percentage	0.0172	0.1910	10
BNI LastFU Complete	vaba_percentage	0.0538	0.2480	10
BNI LastFU Complete	prior_rhizotomy_percentage	-0.0229	0.3020	10
BNI LastFU Complete	prior_srs_percentage	-0.1430	0.0550	10
BNI LastFU Complete	v1_percentage	0.0209	0.6770	8
BNI LastFU Complete	v2_percentage	0.0464	**0.0488**	8

BNI LastFU Complete	v3_percentage	0.0033	0.8610	8
BNI LastFU Complete	v1v2_percentage	0.0191	0.5040	9
BNI LastFU Complete	v2v3_percentage	0.0017	0.9370	9
BNI LastFU Complete	v1v2v3_percentage	-0.0575	0.2530	9
BNI LastFU Complete	interposition_percentage	0.0025	0.7560	10
BNI LastFU Complete	transposition_percentage	-0.0017	0.8210	9
BNI LastFU Adequate	Mean.Age	-0.0135	0.8920	12
BNI LastFU Adequate	Mean.Duration.Pain	0.0046	0.8430	10
BNI LastFU Adequate	Mean.Follow.Up	-0.0098	0.3740	11
BNI LastFU Adequate	male_percentage	-0.0203	0.4730	12
BNI LastFU Adequate	female_percentage	0.0203	0.4730	12
BNI LastFU Adequate	right_percentage	0.0096	0.6770	12
BNI LastFU Adequate	left_percentage	-0.0096	0.6770	12
BNI LastFU Adequate	va_percentage	-0.0182	0.1520	12
BNI LastFU Adequate	ba_percentage	0.0220	0.1540	12
BNI LastFU Adequate	vaba_percentage	0.0555	0.2880	12
BNI LastFU Adequate	prior_rhizotomy_percentage	-0.0022	0.9020	12
BNI LastFU Adequate	prior_srs_percentage	-0.1300	0.1100	12
BNI LastFU Adequate	v1_percentage	0.0006	0.9900	10
BNI LastFU Adequate	v2_percentage	0.0304	0.2100	10
BNI LastFU Adequate	v3_percentage	-0.0015	0.9300	10
BNI LastFU Adequate	v1v2_percentage	0.0110	0.7470	11
BNI LastFU Adequate	v2v3_percentage	0.0045	0.8330	11
BNI LastFU Adequate	v1v2v3_percentage	-0.0411	0.3830	11
BNI LastFU Adequate	retrosigmoid_percentage	-0.0079	0.5920	12
BNI LastFU Adequate	otherapproach_percentage	0.0079	0.5920	12
BNI LastFU Adequate	interposition_percentage	0.0029	0.6890	12
BNI LastFU Adequate	transposition_percentage	-0.0022	0.7550	11
Pain Recurrence	Mean.Age	0.0275	0.7380	13
Pain Recurrence	Mean.Duration.Pain	0.0073	0.7020	11

Pain Recurrence	Mean.Follow.Up	0.0042	0.6430	12
Pain Recurrence	male_percentage	0.0141	0.5630	13
Pain Recurrence	female_percentage	-0.0141	0.5630	13
Pain Recurrence	right_percentage	-0.0073	0.6750	13
Pain Recurrence	left_percentage	0.0073	0.6750	13
Pain Recurrence	va_percentage	0.0171	0.1660	12
Pain Recurrence	ba_percentage	-0.0197	0.1840	12
Pain Recurrence	vaba_percentage	-0.0602	0.2480	12
Pain Recurrence	prior_rhizotomy_percentage	0.0043	0.7840	13
Pain Recurrence	prior_srs_percentage	0.0850	0.2210	13
Pain Recurrence	v1_percentage	-0.0011	0.9800	11
Pain Recurrence	v2_percentage	-0.0354	0.1380	11
Pain Recurrence	v3_percentage	0.0027	0.8720	11
Pain Recurrence	v1v2_percentage	0.0052	0.8540	12
Pain Recurrence	v2v3_percentage	-0.0113	0.5680	12
Pain Recurrence	v1v2v3_percentage	0.0351	0.4140	12
Pain Recurrence	retrosigmoid_percentage	0.0086	0.5580	13
Pain Recurrence	otherapproach_percentage	-0.0086	0.5580	13
Pain Recurrence	interposition_percentage	0.0001	0.9890	12
Pain Recurrence	transposition_percentage	-0.0007	0.9170	11
Salvage Intervention	Mean.Age	0.0788	0.4830	10
Salvage Intervention	Mean.Duration.Pain	0.0236	0.2960	8
Salvage Intervention	Mean.Follow.Up	0.0105	0.3690	9
Salvage Intervention	male_percentage	0.0341	0.2480	10
Salvage Intervention	female_percentage	-0.0341	0.2480	10
Salvage Intervention	right_percentage	-0.0359	0.1790	10
Salvage Intervention	left_percentage	0.0359	0.1790	10
Salvage Intervention	va_percentage	0.0086	0.5850	10
Salvage Intervention	ba_percentage	-0.0087	0.6560	10
Salvage Intervention	vaba_percentage	-0.0359	0.5180	10

Salvage Intervention	prior_rhizotomy_percentage	-0.0177	0.5460	10
Salvage Intervention	prior_srs_percentage	0.0236	0.8700	10
Salvage Intervention	v1_percentage	-0.0325	0.5240	9
Salvage Intervention	v2_percentage	-0.0290	0.2580	9
Salvage Intervention	v3_percentage	-0.0089	0.6390	9
Salvage Intervention	v1v2_percentage	0.0344	0.2960	9
Salvage Intervention	v2v3_percentage	-0.0071	0.7640	9
Salvage Intervention	v1v2v3_percentage	0.0548	0.4240	9
Salvage Intervention	retrosigmoid_percentage	0.0043	0.7760	10
Salvage Intervention	otherapproach_percentage	-0.0043	0.7760	10
Salvage Intervention	interposition_percentage	0.0013	0.8890	10
Salvage Intervention	transposition_percentage	-0.0020	0.8290	9
Overall Complication	Mean.Age	0.0242	0.7540	13
Overall Complication	Mean.Duration.Pain	-0.0023	0.9210	11
Overall Complication	Mean.Follow.Up	0.0013	0.8760	12
Overall Complication	male_percentage	-0.0076	0.7280	13
Overall Complication	female_percentage	0.0076	0.7280	13
Overall Complication	right_percentage	-0.0169	0.4040	13
Overall Complication	left_percentage	0.0169	0.4040	13
Overall Complication	va_percentage	-0.0076	0.5970	12
Overall Complication	ba_percentage	0.0127	0.4980	12
Overall Complication	vaba_percentage	-0.0001	0.9990	12
Overall Complication	prior_rhizotomy_percentage	0.0311	**0.0417**	13
Overall Complication	prior_srs_percentage	0.1104	0.1970	13
Overall Complication	v1_percentage	0.0489	0.1630	11
Overall Complication	v2_percentage	-0.0036	0.8560	11
Overall Complication	v3_percentage	0.0210	0.1200	11
Overall Complication	v1v2_percentage	-0.0310	0.3000	12
Overall Complication	v2v3_percentage	0.0107	0.5800	12
Overall Complication	v1v2v3_percentage	0.0104	0.8190	12

Overall Complication	retrosigmoid_percentage	0.0138	0.2650	13
Overall Complication	otherapproach_percentage	-0.0138	0.2650	13
Overall Complication	interposition_percentage	0.0017	0.8320	12
Overall Complication	transposition_percentage	0.0006	0.9450	11
Transient Complication	Mean.Age	0.0007	0.9940	12
Transient Complication	Mean.Duration.Pain	0.0067	0.8180	10
Transient Complication	Mean.Follow.Up	-0.0035	0.7670	11
Transient Complication	male_percentage	-0.0170	0.5210	12
Transient Complication	female_percentage	0.0170	0.5210	12
Transient Complication	right_percentage	-0.0277	0.3600	12
Transient Complication	left_percentage	0.0277	0.3600	12
Transient Complication	va_percentage	-0.0104	0.5200	12
Transient Complication	ba_percentage	0.0169	0.4110	12
Transient Complication	vaba_percentage	-0.0006	0.9910	12
Transient Complication	prior_rhizotomy_percentage	0.0389	0.0661	12
Transient Complication	prior_srs_percentage	0.0619	0.6170	12
Transient Complication	v1_percentage	0.0536	0.1640	10
Transient Complication	v2_percentage	0.0033	0.8880	10
Transient Complication	v3_percentage	0.0205	0.1890	10
Transient Complication	v1v2_percentage	-0.0251	0.4590	11
Transient Complication	v2v3_percentage	0.0170	0.4510	11
Transient Complication	v1v2v3_percentage	0.0079	0.8860	11
Transient Complication	retrosigmoid_percentage	0.0100	0.4770	12
Transient Complication	otherapproach_percentage	-0.0100	0.4770	12
Transient Complication	interposition_percentage	0.0089	0.2460	12
Transient Complication	transposition_percentage	-0.0051	0.5650	11
Permanent Complication	Mean.Age	0.0956	0.3180	12
Permanent Complication	Mean.Duration.Pain	-0.0169	0.4390	10
Permanent Complication	Mean.Follow.Up	0.0146	0.0867	11
Permanent Complication	male_percentage	0.0378	0.1460	12

Permanent Complication	female_percentage	-0.0378	0.1460	12
Permanent Complication	right_percentage	0.0029	0.9060	12
Permanent Complication	left_percentage	-0.0029	0.9060	12
Permanent Complication	va_percentage	-0.0167	0.1930	12
Permanent Complication	ba_percentage	0.0258	0.0945	12
Permanent Complication	vaba_percentage	0.0072	0.8740	12
Permanent Complication	prior_rhizotomy_percentage	-0.0126	0.6260	12
Permanent Complication	prior_srs_percentage	0.0926	0.3180	12
Permanent Complication	v1v2_percentage	0.0040	0.9150	11
Permanent Complication	v2v3_percentage	-0.0206	0.4000	11
Permanent Complication	v1v2v3_percentage	-0.0047	0.9370	11
Permanent Complication	retrosigmoid_percentage	0.0049	0.7500	12
Permanent Complication	otherapproach_percentage	-0.0049	0.7500	12
Permanent Complication	interposition_percentage	-0.0130	0.0611	12
Permanent Complication	transposition_percentage	0.0127	0.0721	11

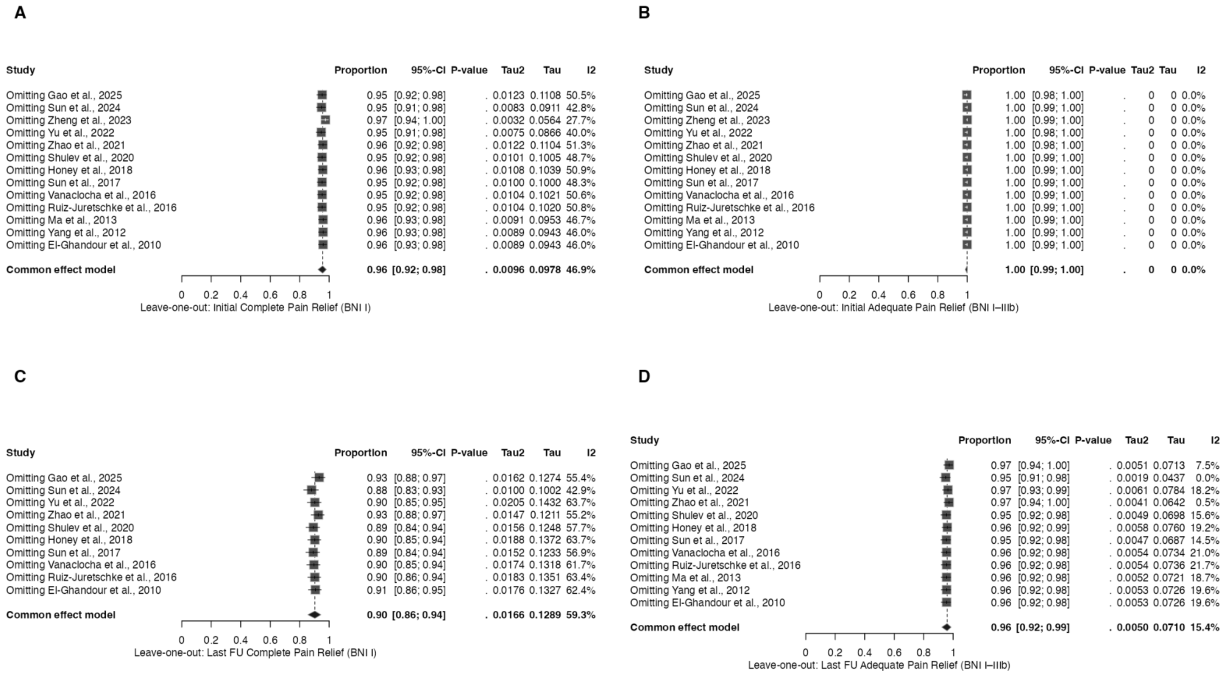

Supplementary Figure 1. Leave-one-out sensitivity analyses of pain outcomes following MVD for VBD-TN: (A) initial complete pain relief, (B) initial adequate pain relief, (C) last follow-up complete pain relief, (D) last follow-up adequate pain relief.

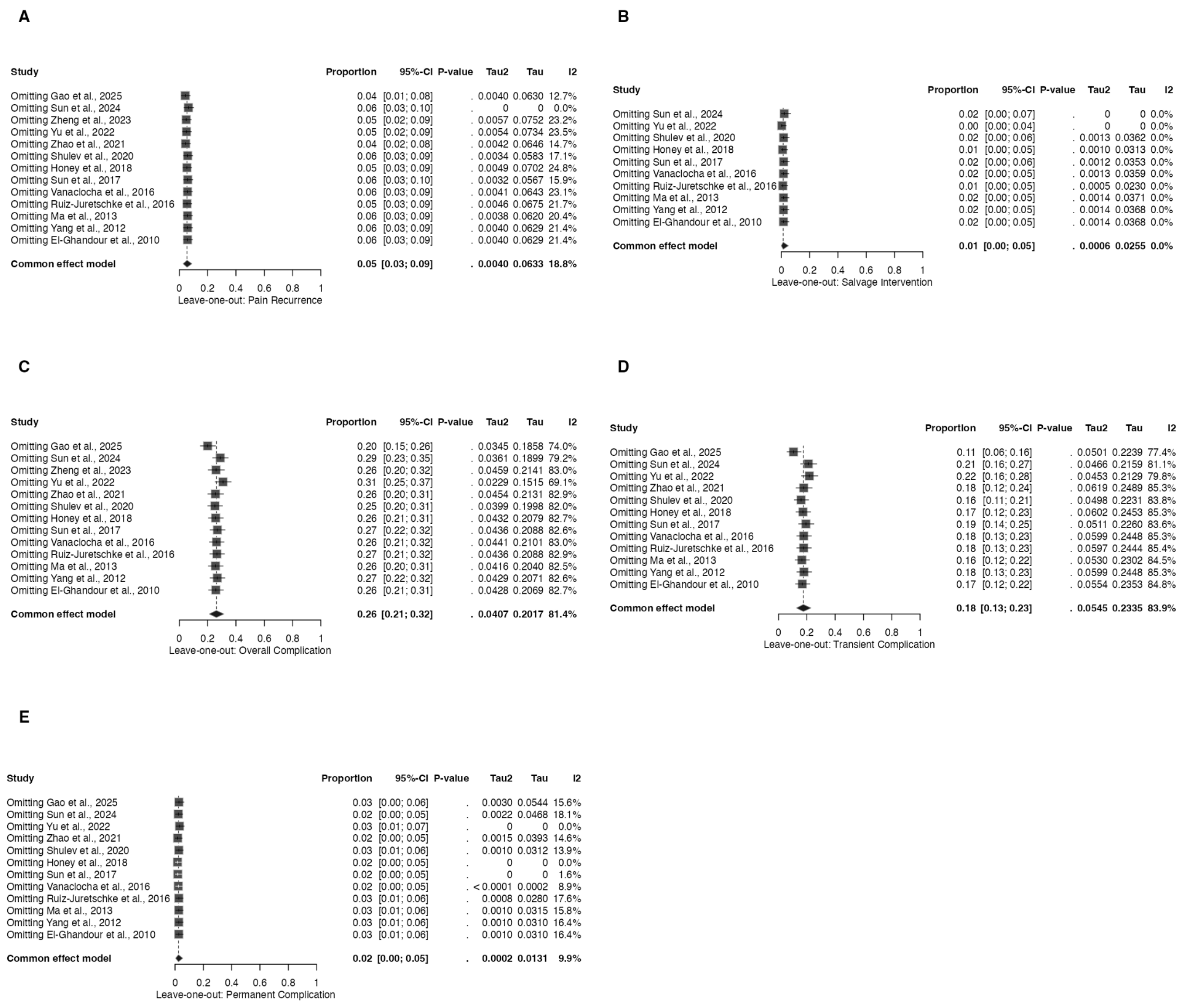

Supplementary Figure 2. Leave-one-out sensitivity analyses of secondary outcomes following MVD for VBD-TN: (A) pain recurrence, (B) salvage interventions, (C) overall complications, (D) transient complications, (E) permanent complications.
